# Several FDA-Approved Drugs Effectively Inhibit SARS-CoV-2 Infection in vitro

**DOI:** 10.3389/fphar.2020.609592

**Published:** 2021-02-05

**Authors:** Hua-Long Xiong, Jia-Li Cao, Chen-Guang Shen, Jian Ma, Xiao-Yang Qiao, Tian-Shu Shi, Sheng-Xiang Ge, Hui-Ming Ye, Jun Zhang, Quan Yuan, Tian-Ying Zhang, Ning-Shao Xia

**Affiliations:** ^1^ State Key Laboratory of Molecular Vaccinology and Molecular Diagnostics, National Institute of Diagnostics and Vaccine Development in Infectious Diseases, School of Life Sciences and School of Public Health, Xiamen University, Xiamen, China; ^2^ Department of Clinical Laboratory, Women and Children’s Hospital, School of Medicine, Xiamen University, Xiamen, China; ^3^ Shenzhen Key Laboratory of Pathogen and Immunity, National Clinical Research Center for Infectious Disease, State Key Discipline of Infectious Disease, Shenzhen Third People’s Hospital, Second Hospital Affiliated to Southern University of Science and Technology, Shenzhen, China; ^4^ School of Public Health, Southern Medical University, Guangzhou, China

**Keywords:** drug screening, SARS-CoV-2, pseudovirus assay, vesicular stomatitis virus, drug combination

## Abstract

To identify drugs that are potentially used for the treatment of COVID-19, the potency of 1403 FDA-approved drugs were evaluated using a robust pseudovirus assay and the candidates were further confirmed by authentic SARS-CoV-2 assay. Four compounds, Clomiphene (citrate), Vortioxetine, Vortioxetine (hydrobromide) and Asenapine (hydrochloride), showed potent inhibitory effects in both pseudovirus and authentic virus assay. The combination of Clomiphene (citrate), Vortioxetine and Asenapine (hydrochloride) is much more potent than used alone, with IC50 of 0.34 μM.

## Introduction

As of September 22, 2020, the COVID-19 pandemic has claimed more than 966,399 lives, but yet effective drug is not available. It is time-consuming to develop vaccines or specific drugs for a disease caused by a novel defined virus like SARS-CoV-2. Re-purposing of approved drugs may be a faster way to find treatment for COVID-19. Verification of drugs that might suppress SARS-CoV-2 by prediction, including drugs against similar virus and broad-spectrum antiviral agents (BSAAs), is time-saving for drug re-purposing at the expense of missing some potential candidates. Integrative, antiviral drug repurposing methods based on big data analysis or molecular docking and molecular dynamics are time-saving and high throughput. However, drugs identified by virtual screening still need to be verified *in vitro* and *in vivo*.

In our previous research, a robust neutralization assay was established based on SARS-CoV-2 S-bearing vesicular stomatitis virus (VSV) pseudovirus and human ACE2-expressing BHK21 cells (BHK21-hACE2) (Xiong et al., 2020). Single-cycle infectious of recombinant VSV-SARS-CoV-2-Sdel18 mimics the entry of SARS-CoV-2. The BHK21-hACE2 cells with high expression level of human angiotensin-converting enzyme 2 (hACE2) need only 6 h to proliferate one generation, which support efficiently infection of pseudovirus and infection of pseudovirus can be detected by fluorescence 12 h after infection, enabling the assay time-saving for high-throughput screening (Xiong et al., 2020). This pseudovirus based assay is suitable for screening drugs that can block the infection of SARS-CoV-2. In this study, the anti-SARS-CoV-2 potentiality of 1403 FDA approved drugs were quantitatively evaluated by the pseudovirus-based assay and the effect of candidate drugs were confirmed using authentic virus assay.

## Results

### Screen for Compounds Could Inhibit the Infection of Vesicular Stomatitis Virus-SARS-CoV-2-Sdel18

**Figure 1:**
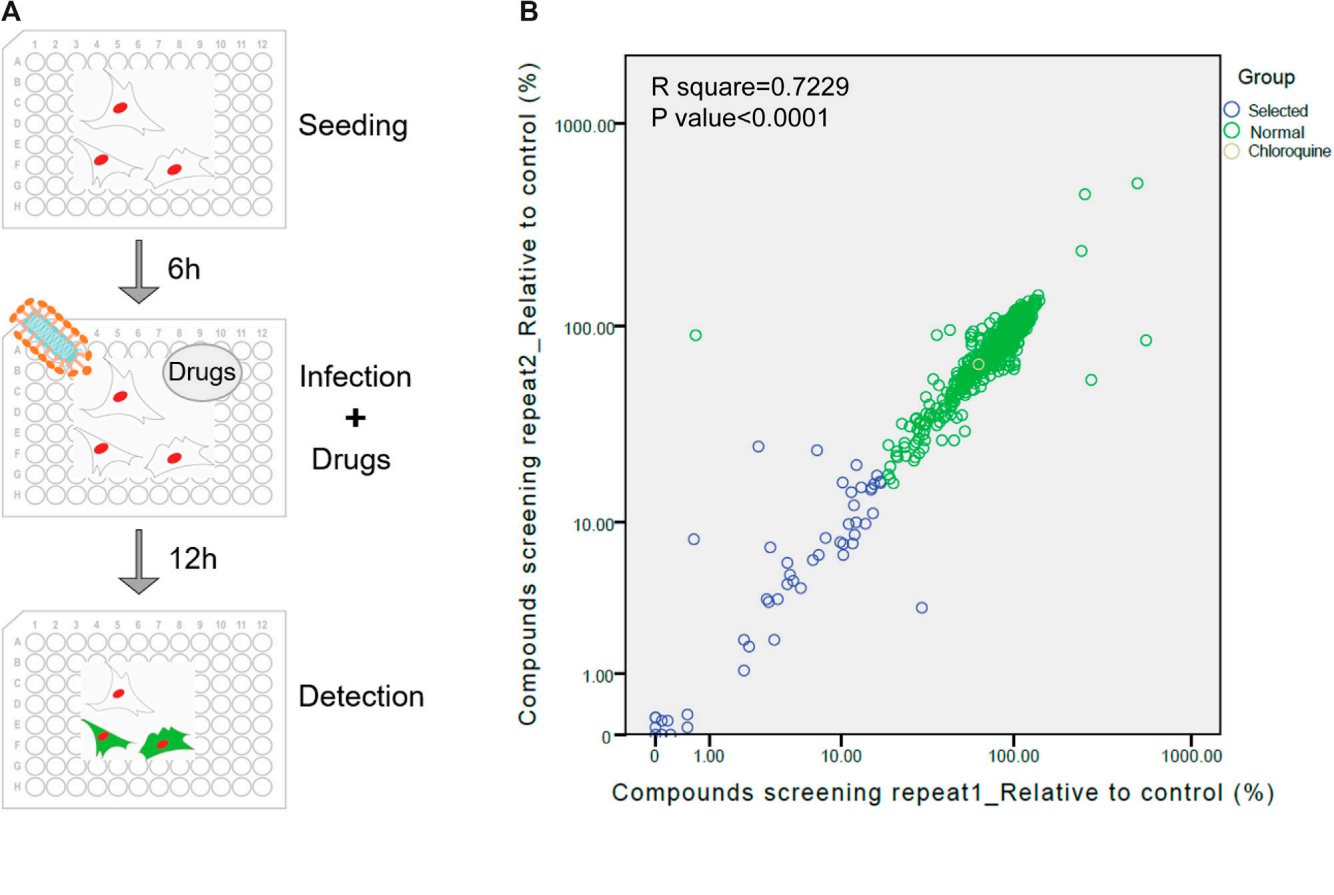
Re-purposing of FDA-approved drugs for inhibiting SARS-CoV-2 infection. **(A)** Schematic diagram of the screening process of the pseudovirus model. **(B)** The first round of screening for compounds could inhibit the infection of VSV-SARS-CoV-2-Sdel18. The abscissa and ordinate respectively indicate two repetitions of screening. Yellow circle: chloroquine. Blue circles: 44 drugs selected for the second round of screening, Green circles: remaing FDA-approved drugs.

The screening procedure was illustrated in Figure 1A and described in methods. The numbers of GFP-positive cells from drug treated wells were counted and divided by the number of infected cells from the well without treatment of drugs to calculate the relative value of infection rate. The results of two repetitions showed that most of drugs did not inhibit viral infection (Figure 1B). Forty-four drugs with relatively better inhibitory effect, whose inhibit ratio were higher than 85% (relative value below 15%) were selected for further validation.

**Figure 2:**
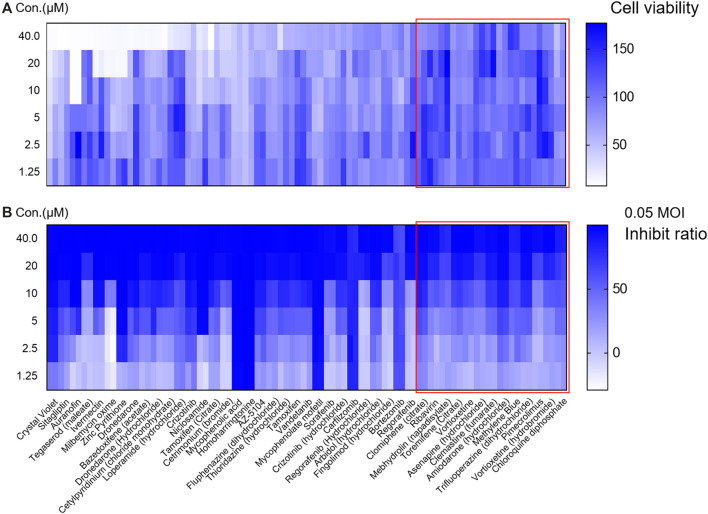
Evaluate the effect and cytotoxicity of the 44 compounds selected in the first round of screening. The 44 compounds and control (chloroquine diphosphate) were seriously diluted to analyze the cytotoxicity **(A)** and effect in inhibiting viral infection **(B)**. Colorbar indicates cell viability **(A)** or inhibiton rate **(B)**.

In the second round of screening, the effect of inhibiting viral infection and cell cytotoxicity in different concentration conditions were both evaluated (Figure 2). Among them, 32 drugs were excluded due to cytotoxicity (cell viability were lower than 80% when treated with compounds at concentration of 40 μM or cell viability were lower than 85% when compounds were used at concentration of 20 μM). Twelve drugs were selected for analysis of specificity to VSV-SARS-CoV-2-Sdel18 and verification by authentic SARS-CoV-2 assay.

### The Specificity of Selected Compounds for Vesicular Stomatitis Virus-SARS-CoV-2-Sdel18

**Figure 3:**
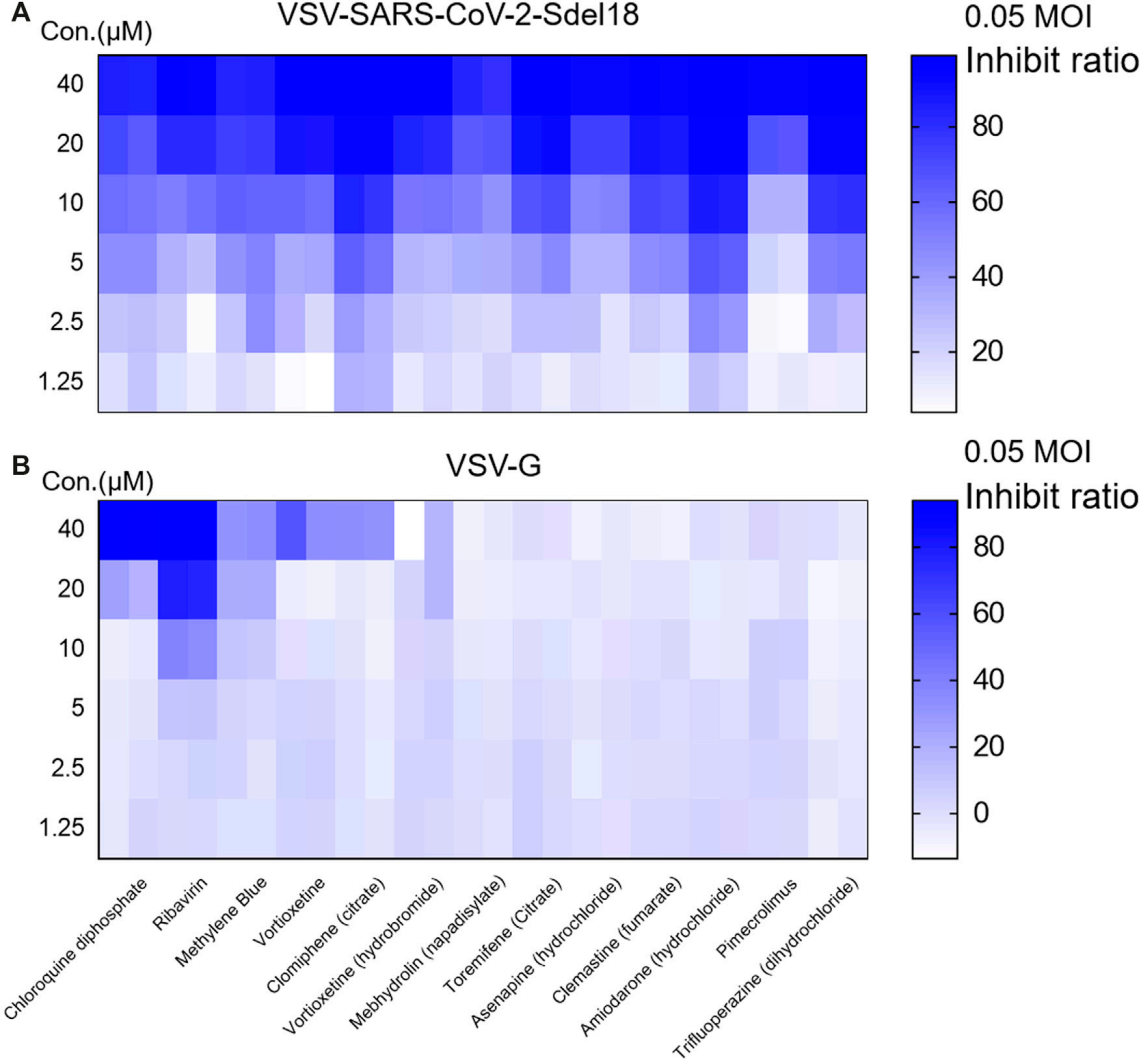
Screening for the compounds specially block the spike protein of SARS-CoV-2 mediated viral entry. The inhibitory effect to VSV-SARS-CoV-2-Sdel18 **(A)** and VSV-G **(B)** of 13 seleted compounds was evaulated to exclude drugs inhibit infection or expression of VSV-G. Colorbar indicates inhibiton rate.

To verify whether these selected drugs act on spike protein of SARS-CoV-2 on the pseudovirus or the VSV backbone, we evaluated the inhibitory effect of these compounds on VSV-G (The sequence of GFP was inserted into the genome of VSV, so that the infection of VSV could be indicated by green fluorescence.). Ribavirin exhibited significant inhibitor effects on VSV-G, whereas no obvious effect was noted for other compounds (Figure 3).

### The Effect of Selected Compounds in Pseudovirus Assay and Authentic Virus Assay

**Figure 4:**
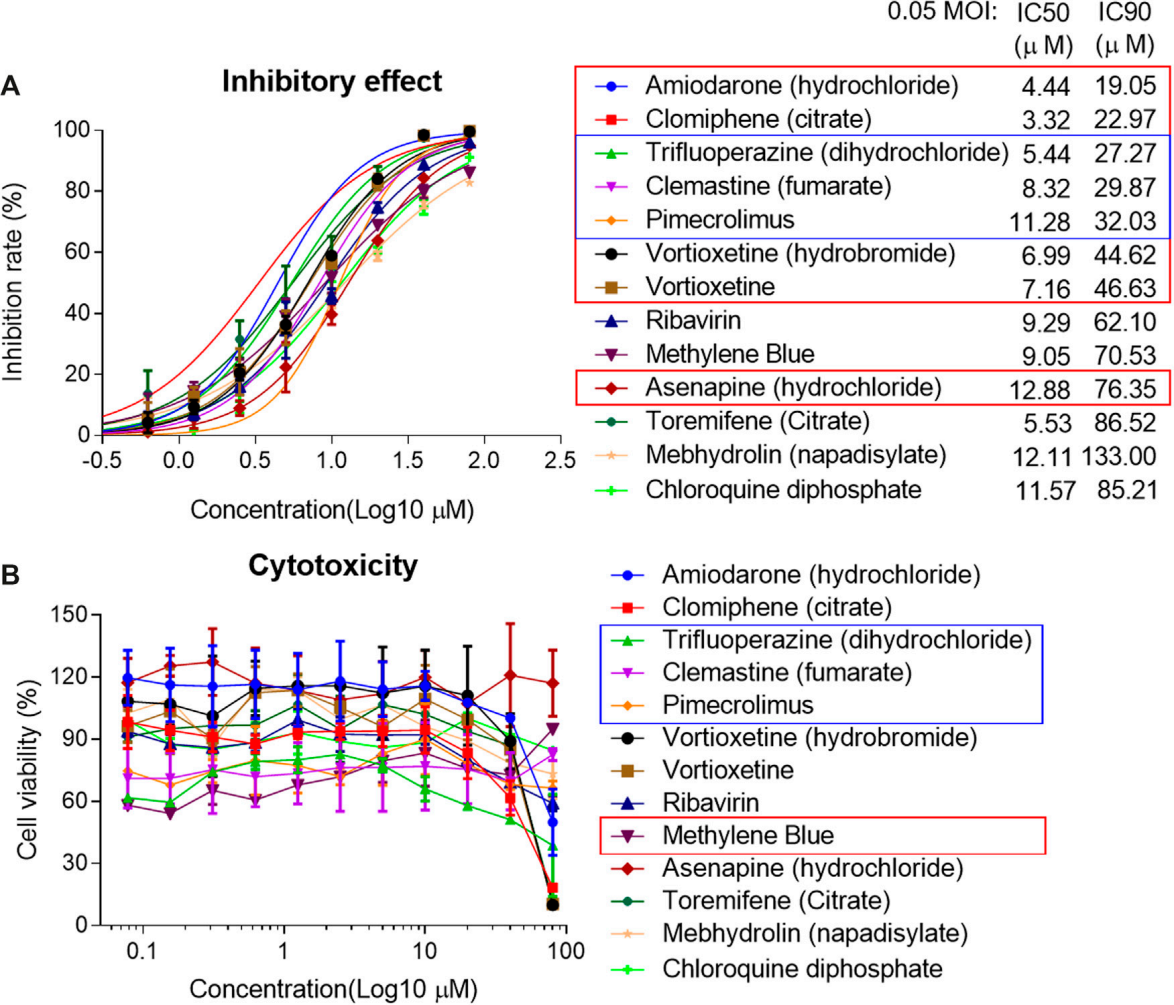
Analyze the inhibitory effect of 12 selected compounds in VSV-SARS-CoV-2-Sdel18 pseudovirus assay. The 12 compounds and control (chloroquine diphosphate) were seriously diluted to analyze the effect in inhibiting viral infection **(A)** and cytotoxicity **(B)**. The IC50 and IC90 were calculated with non-linear regression.

**Figure 5:**
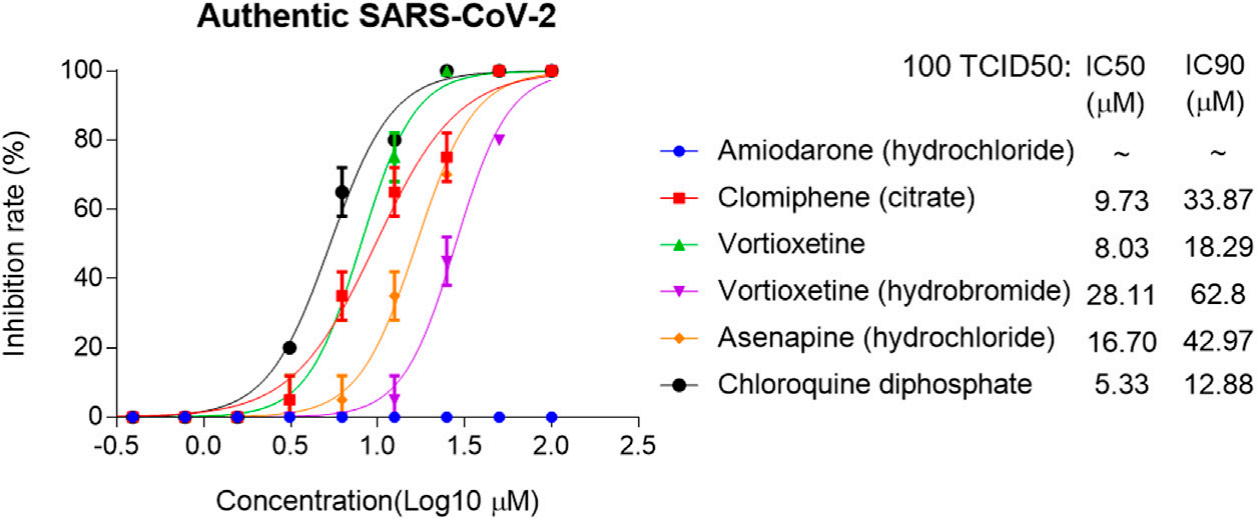
Analyze the inhibitory effect of five selected compounds in authentic SARS-CoV-2 assay. The IC50 and IC90 were calculated with non-linear regression.

The IC50 and IC90 for VSV-SARS-CoV-2-Sdel18 pseudovirus were further analyzed (Figure 4). Of the 12 compounds that selected in the second round of screening, seven drugs could inhibit viral infection with IC90 lower than 50 μM, including Amiodarone (hydrochloride), Clomiphene (citrate), Trifluoperizine (dihydrochloride), Clemastine (fumarate), Pimecrolimus, Vortioxetine (hydrobromide) and Vortioxetine. Trifluoperizine (dihydrochloride), Clemastine (fumarate) and Pimecrolimus showed more serious cytotoxic than other drugs. Although the inhibitory effect of Asenapine (hydrochloride) is not as good as the seven compounds mentioned previously, it has the lowest cytotoxicity. Even when used at the concentration of 100 μM, no obvious cell cytotoxicity was observed. Considering the inhibitory effect and cytotoxicity, five compounds inhibited the infection of VSV-SARS-CoV-2-Sdel18 pseudovirus specifically, including Clomiphene (citrate), Amiodarone (hydrochloride), Vortioxetine, Vortioxetine (hydrobromide) and Asenapine (hydrochloride), were selected and the function of these compounds was confirmed using authentic SARS-CoV-2 assay (Figure 5). Among them, the inhibitory effects of Clomiphene (citrate) and Vortioxetine were comparable to Chloroquine diphosphate *in vitro*, while Vortioxetine (hydrobromide) and Asenapine (hydrochloride) were slightly less effective. Whereas Amiodarone (hydrochloride) inhibited the infection of pseudovirus efficiently with IC50 around 4.44 μM, but it showed no effect on authentic SARS-CoV-2 virus infection even used at a concentration of 100 μM.

### The Potential Applications in Prophylaxis and Combination Therapy

We treated the cell with pseudovirus and different drug combinations. The drug combinations were added either at the same time of pseudovirus infection or 6 h pre-infection (Table 1). The combination of Clomifene (citrate), Vortioxetine and Asenapine (hydrochloride) showed best effect when used both at the time of infection and pre-infection, with IC50 about 1.93 and 0.34 μM respectively. The combination of Clomifene (citrate) and Vortioxetine had a comparable effect, with IC50 about 2.36 and 0.69 μM respectively. The combination of drugs decreases the concentration of each drug required to block virus infection, which may reduce the side effects of drugs. However, it remains to be evaluated whether these drugs can be used together *in vivo*.

**TABLE 1:** The inhibitory potency of combination of drugs.

Drug	Pre-treatment			Co-treatment		
	IC50 (μM)	IC90 (μM)	CC50 (μM)	IC50 (μM)	IC90 (μM)	CC50 (μM)
Clo + Vor	0.69	4.81	14.47	2.36	11.80	23.55
Clo + Ase	1.60	10.39	21.39	3.71	20.06	36.20
Vor + Ase	2.08	11.06	14.12	3.52	17.47	28.00
Clo + Vor + Ase	0.34	5.01	14.67	1.93	9.42	16.83
Clo + CQ	2.57	12.81	28.01	7.32	17.99	37.98
Vor + CQ	3.03	13.00	∼18.93	5.39	17.87	25.69
Ase + CQ	5.73	55.30	128.00	12.70	58.87	Na
Clo	2.94	10.16	24.94	9.53	19.57	54.35
Vor	3.00	13.83	28.53	6.77	22.28	27.05
Ase	17.69	127.10	Na	28.13	117.60	Na
CQ	9.27	35.22	349.10	27.60	106.20	Na

“Clo” means Clomiphene (citrate), “Vor” means Vortioxetine, “Ase” means Asenapine (hydrochloride) and “CQ” means Chloroquine diphosphate. “Pre-treatment” means cell was treated with drugs 6 h before infection, while “Co-treatment” means cells were treated with drugs at the time of infection. IC50, IC90 and CC50 were calculated using prism software (GraphPad). “na” means the value can’t be calculated. MOI = 0.1.

## Discussion

Thousands of clinical trials have been initiated to establish evidence around investigational drugs and vaccine candidates. There are currently no approved vaccines against SARS-CoV-2 for commercial use, except for two approved for early or limited use. COVID-19 vaccine candidate of CanSino and CanSino have been approved, but only for military use or medical workers. The Food and Drug Administration (FDA) has not fully approved any medication for treating people infected with SARS-CoV-2. Some drugs with good effects in clinical use are granted emergency use authorizations for certain patients hospitalized with COVID-19, such as dexamethasone and remdesivir. Dexamethasone, a cheap and widely available steroid, cut deaths by one-third among patients critically ill with COVID-19 in a large trial (Horby and Landrain, 2020). Dexamethasone can alleviate the overreaction of the immune system, which is a main cause of severe cases and fatalities (Lammers et al., 2020). Remdesivir, an investigational nucleotide analog with broad-spectrum antiviral activity by inhibiting viral replication, also showed clinical improvement. These two drugs have different mechanisms of action, the combination may be complimentary.

Both dexamethasone and remdesivir act on the steps after viral infection. The combination of drugs act on viral entry in addition may benefit further. The effect of chloroquine and hydroxychloroquine has attracted much attention. Several *in vitro* studies reported antiviral activity of chloroquine and hydroxychloroquine against SARS-CoV-2. However, this drug provided no additional benefit compared to placebo control for the treatment of COVID-19 in hospitalized patients (Frie and Gbinigie, 2020). In addition, cardiomyopathy and heart rhythm disturbances caused by treatment with chloroquine have been reported and Risambaf et al. raise concerns about the risk of toxicity to liver and kidney caused by chloroquine and hydroxychloroquine when they are used to treat COVID-19 (Cubero et al., 1993; Costedoat-Chalumeau et al., 2007; Rismanbaf and Zarei, 2020). Drugs that inhibit the infection of SARS-CoV-2 with higher efficiency and lower side effect may be alternative for the treatment of COVID-19.

Drugs that block the infection of SARS-CoV-2 may alleviate disease progression, protect health care workers and other populations at high risk of infection. In addition, drugs inhibit viral infection might be used in combination with drugs that inhibit viral replication reported previously. In this research, an efficient VSV-SARS-CoV-2-Sdel18 pseudovirus model was applied to identify candidates that can inhibit infection of SARS-CoV-2 from 1,403 approved drugs. Five drugs, which haven’t been identified before, showed comparable or superior inhibitory effect to chloroquine in this model. The effect was also confirmed using authentic SARS-CoV-2 assay and four of them can also inhibit the infection of authentic SARS-CoV-2 virus.

Clomifene Citrate is a selective estrogen receptor modulator and a non-steroidal fertility medicine. It has a long history of use since 1967 and has the advantages of oral availability, good safety, and tolerability profiles. Johansen et al. identified Clomiphene as potent inhibitors of Ebola virus infection by performing an *in vitro* screen of FDA and ex-US-approved drugs. This drug showed EC50 values of 11 and 3.8 μM against the two strains EBOV-95 and EBOV-76, respectively, and a 90% of survival benefit for infected mice. It may inhibit Ebola virus through inducing accumulation of cholesterol in endosomal compartments and blocking the release of viral genome to cytoplasm (Johansen et al., 2013; Wrensch et al., 2014; Nelson et al., 2016). The viral entry of SARS-CoV-2 includes the endocytosis of enveloped viral particle, priming of spike protein by protease, fusion between viral and cellular membranes and release of viral genome, which is similar to Ebola virus (Simmons et al., 2004; Burkard et al., 2014; Hoffmann et al., 2020). Therefore, the Clomiphene may impair SARS-CoV-2 infection via the same pathway as Ebola virus.

Vortioxetine is an antidepressant drug that is used to treat major depressive disorder in adults. Vortioxetine was safe and well tolerated, it was approved in 2013 (Baldwin et al., 2016). So far, no previous study described its antiviral roles. It is reported that sever COVID-19 patients have a high probability of suffering from mental illness. Recently, another antidepressant drug fluvoxamine is evaluated for the potential to treat COVID-19 by researchers from the Washington University School of Medicine, because the drug may prevent an overreaction of the immune system called cytokine storms, which could result in life-threatening organ failure. The antiviral mechanism of Vortioxetine remains unknow. However, it may bring physical and psychological benefits for COVID-19 patients.

Asenapine is an atypical antipsychotic drug which has been approved by the US Food and Drug Administration for the treatment of schizophrenia in adults and the treatment of acute manic or mixed episodes of bipolar I in both adult and pediatric populations. Asenapine is a tetracyclic drug with antidopaminergic and antiserotonergic activity with a unique sublingual route of administration and has been approved since 2009. It showed less cytotoxicity in this study comparing to other drugs that could inhibit the infection of SARS-CoV-2. Notably, although we have evaluated the effect of these candidate drugs in two different *in vitro* model and the combination of these drugs didn’t show obvious cytotoxicity *in vitro*, the effect and safety *in vivo* still remain to be confirmed.

Several drug screenings for COVID-19 have been performed before and identified some candidate drugs, for example, Yadi Zhou et al. prioritized 16 potential anti-HCoV repurposable drugs (e.g., melatonin, mercaptopurine, and sirolimus) by using network proximity analyses of drug targets and HCoV–host interactions in the human interactome, drug target proteins select by Rameez Jabeer Khan et al. were screened against an in-house library of 123 antiviral drugs, they proposed that Raltegravir, Paritaprevir, Bictegravir and Dolutegravir are excellent lead candidates for these crucial proteins and they could become potential therapeutic drugs against SARS-CoV-2, Laura Riva et al. discovered SARS-CoV-2 antiviral drugs through large-scale compound repurposing by authentic SARS-CoV-2 assay (Dyall et al., 2014; Khan et al., 2020; Riva et al., 2020; Weston et al., 2020; Zhou et al., 2020). The focus of these studies varies and shed light on the treatment of COVID-19. The hits screened out from our study were different from other studies. The combination of drug candidates obtained by different screening strategies may have synergistic effect. The screening assay based on the single-cycle infectious VSV-SARS-CoV-2-Sdel18 has its advantage from the practical perspectives-manipulation in BSL-2. However, this assay also has disadvantages. Firstly, it may not be able to screen out compounds that can specifically target the steps of SARS-CoV-2 life cycle after viral entry; secondly, it may screen out compounds that inhibit the VSV, but not SARS-CoV-2. To address the second weakness, the specificity of hits out from the pseudovirus assay were confirmed using VSV-WT and the authentic SARS-CoV-2 assay. The candidates proposed in this study mainly function on inhibiting the viral entry, they could be combined with drugs act on other pathways, for example, combined with Remdesivir that inhibit replication of virus. Another limitation of the model is that hACE2 overexpressing BHK21 cell was derived from hamster and Vero cell supporting the infection of authentic SARS-CoV-2 was from African green monkey. The effects of candidate compounds in human cells also needs to be further verified.

In summary, our study identified four FDA-approved drugs that have the potential to suppress SARS-CoV-2 infection. The robust assay based on VSV-SARS-CoV-2-Sdel18 pseudovirus screened out the potential drugs with high efficiency, then the inhibitory effect was confirmed by authentic SARS-CoV-2 assay. The inhibitory effect of Vortioxetine and Clomifene is superior and the mechanism of these drugs seems different from Chloroquine. The combination of Clomifene (citrate), Vortioxetine and Asenapine (hydrochloride) greatly decreases the IC50/IC90 of blocking virus infection. The clinical safety of these compounds has been evaluated and the availability of pharmacological data are expected to enable rapid preclinical and clinical evaluation for treatment of COVID-19. Based on the existing clinical results, it seems that it is difficult for one particular drug alone to significantly benefit COVID-19 patients, and combination therapy is more likely to make the patient recover faster. This work identified novel drugs that suppress the infection of virus and provided more candidates for post-exposure prophylaxis and combination therapies. Notice that no test *in vivo* has been conducted and the mechanism of these compounds also remains unknown. More researches are required to support the clinical application of these drugs for treatment of COVID-19.

## Materials and Methods

### Cells and Samples

Vero-E6 [American Type Culture Collection (ATCC), CRL-1586], Vero (ATCC, CCL-81), BHK21-hACE2 (Xiong et al., 2020) cells were maintained in high glucose DMEM (SIGMA-ALDRICH) supplemented with 10% FBS (GIBCO), penicillin (100 IU/ml), streptomycin (100 μg/ml) in a 5% CO_2_ environment at 37°C and passaged every 2 days. In addition, the culture medium of BHK21-hACE2 contains puromycin (2 μg/ml). The FDA-approved drug library, including 1,403 compounds (10 mM DMSO solutions, MCE, HY-LD-000001083), and Chloroquine diphosphate were bought from MedChemExpress (MCE, HY-17589).

### Pseudovirus-Based Assay

VSV pseudovirus carrying truncated spike protein of SARS-CoV-2, named VSV-SARS-CoV-2-Sdel18 virus, was packaged as previously described (Xiong et al., 2020). VSV-G was prepared in similar way (Whitt, 2010). In the first round of screening, all compounds were diluted to 20 μM and mixed with VSV-SARS-CoV-2-Sdel18 virus, the volume of diluted compounds and virus are 80 and 20 μL respectively. Each dilution repeated twice. Added 80 μL final mixture, which containing compounds (16 μM) and pseudovirus (MOI = 0.05), to pre-seeded BHK21-hACE2. After 12 h incubation, fluorescence images were obtained by ImmunSpot@S5 UV Analyzer (Cellular Technology Limited) or Operetta CLS (PerkinElmer). For quantitative determination, the numbers of GFP-positive cell for each well were counted to represent infection performance. The reduction (%) in GFP-positive cell numbers was calculated to show the inhibitory effect of compounds. In the second round of screening, selected compounds were diluted to 50 μM, then serial two-fold dilutions are used to prepare diluted analytes. 80 μL diluted compounds were mixed with 20 μL VSV-SARS-CoV-2-Sdel18 or VSV-G and the mixture were added to pre-seeded BHK21-hACE2. The results were obtained as described previously. To analysis the IC50 of selected compounds, the compounds were diluted to 100 μM as the first work concentration and 0.098 μM as the smallest concentration. Still mixed 80 μL diluted compounds with 20 μL VSV-SARS-CoV-2-Sdel18 virus. The remaining procedures were same as previous assay. The cytotoxicity of compounds was analyzed by Cell Counting Kit-8 (CCK-8, MCE). To evaluate the effect of drug combinations, the drugs were also diluted to 100 μM (the concentration of each drugs is 100 μM in mixture) and prepared serious dilutions. To evaluate the potential of applying these drugs in prophylaxis, the cell was pre-treated with 80 μL diluted drugs, 6 h later, add 20 μL virus to the culture medium (MOI = 0.1). In combination therapy, the combos were prepared in an equal molar ratio.

### Authentic SARS-CoV-2-Based Assay

Vero cells were seeded 24 h before the infection in a 96-well plate (Costar). On the day of infection, the cells were washed twice with PBS. Candidate drugs were diluted 2-fold seriously by medium supplemented with 2% FBS (GIBCO), penicillin (100 IU/ml), streptomycin (100 μg/ml). Each drug was evaluated by diluting 14 gradients, with each gradient double repeats. Aliquots (40 μL) of diluted drugs (200 μM as initial concentration) was added to 40 μL of cell culture medium containing 100 times the tissue culture infective dose (TCID50) of the BetaCoV/Shenzhen/SZTH-003/2020 strain virus (GISAID access number: EPI_ISL_406594) on a 96-well plate in duplicate and incubated at 37°C for 2 h in CO_2_ 5% vol/vol. After incubation, virus drugs mix was then added to cells in 96-well plates and plates were incubated at 37°C with microscopic examination for cytopathic effect after a 5-days incubation. Ten fields of view were randomly selected for each repetition and cytopathic effect was quantified by the number of fields present with CPE. For example, if CPE was observed in seven of ten fields, which mean the cytopathic effect was 70%. The complete absence of cytopathic effect in an individual culture well was defined as protection. The values of IC50 were calculated using prism software (GraphPad).

### Statistic

The relative value or inhibition rate of candidate drugs were calculated according to the decrease of GFP positive cell number (for pseudovirus-based assay) or cytopathic effect (for authentic SARS-CoV-2-based assay). The IC50 (the half maximal inhibitory concentration) and IC90 (the concentration for the 90% of the maximum inhibition) values were calculated with non-linear regression, i.e. log(inhibitor) vs. normalized response—Variable slope or log (agonist) vs. response—Find EC anything using GraphPad Prism 7.00 (GraphPad Software, Inc., San Diego, CA, United States).

## Data Availability Statement

The raw data supporting the conclusions of this article will be made available by the authors, without undue reservation.

## Author Contributions

HX, TZ, QY and NX had full access to all of the data in the study and take responsibility for the integrity of the data and the accuracy of the data analysis. Study concept and design: TZ, QY and NX. Acquisition of data: HX, JC, JM, XQ, TS. CS performed the authentic virus assay. Analysis and interpretation of data: HX, JC, TZ. Drafting of the manuscript: JC, TZ, HX. Critical revision of the manuscript for important intellectual content: SG, JZ. Study supervision: TZ, QY and NX. All authors listed have made a substantial, direct, and intellectual contribution to the work and approved it for publication.

## Funding

This work was supported by the National Natural Science Foundation of China (81702006), Shenzhen Science and Technology Innovation Commission for Research and Development Project (Grants JCYJ20190809183205622).

## Conflict of Interest

The authors declare that the research was conducted in the absence of any commercial or financial relationships that could be construed as a potential conflict of interest.
